# OTC-NET: A Multimodal Method for Accurate Diagnosis of Ovarian Cancer in O-RADS Category 4 Masses

**DOI:** 10.3390/cancers17213466

**Published:** 2025-10-28

**Authors:** Peizhong Liu, Yidan Ruan, Yuling Fan, Ping Li, Zhuosheng Liu, Shengjie Wu, Xinying Zheng, Xiuming Wu, Yiting Liu, Shunlan Liu

**Affiliations:** 1 College of Engineering, Huaqiao University, Quanzhou 362021, China; pzliu@hqu.edu.cn (P.L.);; 2 College of Medicine, Huaqiao University, Quanzhou 362021, China; ryd7777@163.com (Y.R.);; 3 Department of Obstetrics and Gynecology, Quanzhou First Hospital Affiliated to Fujian Medical University, Quanzhou 362000, China; 4 Department of Ultrasound Medicine, The Second Affiliated Hospital of Fujian Medical University, Quanzhou 362000, China; 5 Department of Ultrasound, Quanzhou First Hospital Affiliated to Fujian Medical University, Quanzhou 362000, China; 6 College of Medicine, Shenzhen University, Shenzhen 518055, China

**Keywords:** ovarian lesions, deep learning, ultrasound, O-RADS, radiologists

## Abstract

**Simple Summary:**

Ovarian cancer is the most lethal malignancy of the female reproductive system. Classification of benign and malignant ovarian masses before surgery is crucial for appropriate treatment. O-RADS category 4 ovarian masses often present atypical features, posing significant challenges to radiologists. In this study, we developed a model named OTC-NET, which integrates ultrasound images with clinical information to better identify O-RADS 4 ovarian masses. Compared with individual CNN models and radiologists of varying experience levels, OTC-NET demonstrated higher accuracy and greater stability in detecting malignant lesions. Moreover, after radiologists used OTC-NET as an assistive tool, their diagnostic accuracy further improved. These findings suggest that OTC-NET can help clinicians make more accurate decisions and serve as a valuable aid in addressing the diagnostic challenges of ultrasound-based assessment.

**Abstract:**

Background: Ovarian cancer is the deadliest female reproductive malignancy. Accurate preoperative differentiation of benign and malignant ovarian masses is critical for appropriate treatment. O-RADS category 4 lesions present a wide range of malignant risk, challenging radiologists. Ultrasonic images are the primary focus of current deep learning models, with no consideration for clinical data. Methods: We proposed OTC-NET, a model that uses multimodal data for classification, which combines ultrasound images and clinical information to improve the classification ability of O-RADS 4 ovarian masses. Results: OTC-NET outperforms seven deep learning models and three radiologists of varying experience, with AUC significantly higher than junior (*p* < 0.001), intermediate (*p* < 0.01), and senior (*p* < 0.05) radiologists. Additionally, OTC-NET–assisted diagnosis notably improves AUC and accuracy of junior and senior radiologists (*p* < 0.05). Conclusions: These results indicate that OTC-NET provides superior diagnostic accuracy and has strong potential for clinical application.

## 1. Introduction

Ovarian cancer is one of the most common malignant tumors of the female reproductive system and remains a leading cause of female mortality [1,2]. Accurate differentiation of ovarian masses as benign or malignant is essential for surgical planning, personalized treatment, and avoiding overtreatment [3]. Currently, ultrasound (transvaginal or transabdominal) is the most widely used imaging modality for evaluating ovarian lesions due to its accessibility, non-invasiveness, and low cost [4].

To standardize consistency in ultrasound evaluation, the American College of Radiology (ACR) introduced the Ovarian-Adnexal Reporting and Data System (O-RADS) in 2020 for risk management of lesions. Based on standardized terminology and data-driven risk scores, the system classifies ovarian lesions into six categories: O-RADS 0 (incomplete evaluation), O-RADS 1 (physiologic, normal premenopausal ovary), O-RADS 2 (almost certainly benign, risk < 1%), O-RADS 3 (low risk, 1–<10%), O-RADS 4 (intermediate risk, 10–<50%), and O-RADS 5 (high risk, ≥50%) [5,6]. Adnexal lesions lacking typical benign features are often classified as O-RADS 4 [7]. Accurate classification of O-RADS 4 lesions poses significant diagnostic challenges for radiologists. Moreover, the accuracy of ultrasound diagnosis is influenced by the experience level of the interpreting physician [8,9]. In addition to the O-RADS system, clinical practice also employs other approaches, such as the Risk of Malignancy Index (RMI) and the ADNEX model, which combine imaging features with serum biomarkers (e.g., CA125) for risk assessment [4,10]. However, for complex and atypical O-RADS 4 lesions, these methods still suffer from limited discriminatory power, high operator dependence, and insufficient stability.

In recent years, deep learning has made remarkable progress in medical image analysis [11,12], with multiple studies demonstrating that its performance in classifying ovarian lesions as benign or malignant can match or even surpass that of experienced radiologists [13,14,15,16,17]. However, the atypical nature of O-RADS 4 lesions increases the difficulty of recognition and classification. The effectiveness of existing methods in this context remains unclear. Moreover, most current approaches focus solely on imaging features while neglecting patients’ clinical information, limiting their applicability and generalizability in real-world clinical practice. To address these limitations, this study proposes a multimodal approach, OTC-NET, for classifying O-RADS 4 ovarian lesions. The method combines advanced image classification networks with a random forest algorithm and was validated on 518 cases. Additionally, this study explored the potential of OTC-NET as a diagnostic aid to enhance radiologists’ performance.

## 2. Materials and Methods

### 2.1. Study Population

**Figure 1:**
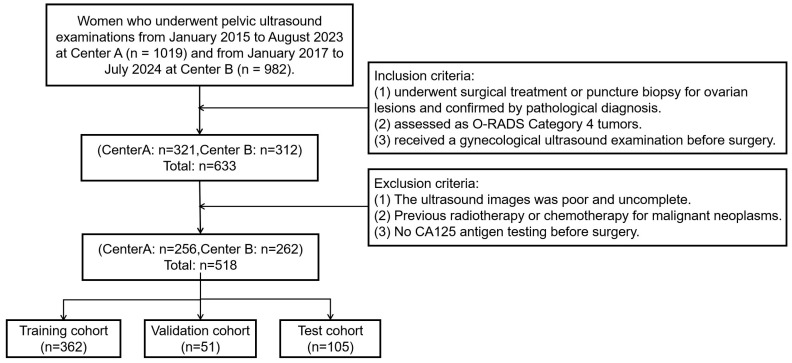
The flowchart of patient enrollment, inclusion, and exclusion criteria, and partitioning of datasets.

The study retrospectively collected data from 518 female patients at two Class A tertiary hospitals in Fujian Province, China, between January 2015 and July 2024. The inclusion criteria were as follows: (1) underwent surgical treatment or puncture biopsy for ovarian lesions and confirmed by pathological diagnosis. (2) assessed as O-RADS Category 4masses by ultrasound radiologists according to the O-RADS lexicon white paper. (3) received a gynecological ultrasound examination before surgery. The exclusion criteria were as follows: (1) The ultrasound images were poor and incomplete. (2) Previous radiotherapy or chemotherapy for malignant neoplasms. (3) No CA125 antigen testing before surgery. Collected data included ultrasound images and clinical information such as age, lesion size, and menopausal status. Figure 1 shows the flowchart of patient enrollment, inclusion, and exclusion criteria, and partitioning of datasets.

**Figure 2:**
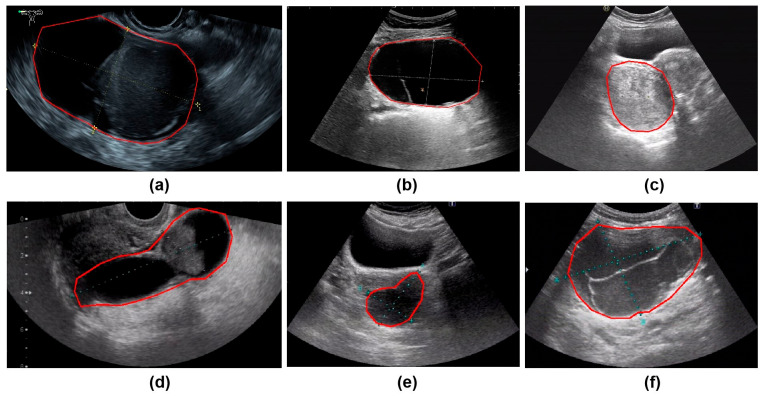
The parts circled in red in the image represent the lesions we labelled. (**a**) A 42-year-old patient with a serous cystadenoma. (**b**) A 35-year-old patient with a mature cystic teratoma. (**c**) A 48-year-old patient with a borderline mucinous tumor. (**d**) A 52-year-old patient with high-grade serous adenocarcinoma. (**e**) A 65-year-old patient diagnosed with an ovarian fibroma; (**f**) A 48-year-old patient with an adult-type granulosa cell tumor.

All ultrasound images were obtained from the Picture Archiving and Communication Systems (PACS). Images were acquired using abdominal probes with a frequency range of 1–6 MHz and transvaginal probes with a frequency range of 2–9 MHz. For each patient, the images representing the largest lesion diameter and most complex morphology were selected. Figure 2 provides representative examples of selected cases from this study. Senior radiologists precisely annotated tumor location and boundaries using the Labelme tool (Version 4.5.9, Cambridge, MA, USA) [18]. The lesion regions were then manually cropped and saved as separate images, which were converted to grayscale to standardize the input format for model training.

Using a random seed based on unique patient identifiers, the 518 patients were randomly assigned to the training, validation, and test sets at a 7:1:2 ratio. Among them, 362 patients with 1133 images were assigned to the training set, 51 patients with 182 images to the validation set, and 105 patients with 302 images to the test set. All experiments, including model training (with data augmentation), model testing, the training and testing of comparison models, and testing by radiologists, were strictly conducted based on this data split to ensure data consistency. The training set was used exclusively to train the model. The validation set was employed during training solely to optimize model parameters. The test set was reserved strictly for evaluating the model’s performance.

This study was approved by the ethics committee of the participating hospital (No. Quanyilun [2024] k246). Since the cases in all datasets were collected retrospectively and de-identified, informed consent was waived. This study adheres to the ethical standards of the 1964 Declaration of Helsinki (2013 revision) and its subsequent amendments.

### 2.2. OTC-NET Network Architecture

OTC-NET is a two-stage classification network designed to integrate ultrasound images and clinical information to predict the benign or malignant status of ovarian masses. The first stage focuses on image-based prediction. Three image classification models (DenseNet201, ResNet34, MobileNet_V2) independently predict all images of each patient [19,20,21]. The prediction probabilities of all images for each case are averaged. If the average probability exceeds 0.5, the case is classified as “malignant”, otherwise as “benign”.

The second stage incorporates clinical features through a Random Forest classifier [22]. Random Forest (RF) is an ensemble learning method. It uses multiple decision trees. RF can reduce model variance and prevent overfitting, which is useful for medical datasets that are small and highly heterogeneous. RF also provides feature importance measures, which help interpret the model and identify the key variables for classifying benign and malignant ovarian masses. The image-based prediction results from the first stage are combined with clinical information, including patient age, menopausal status, maximum lesion diameter, and CA125 levels, forming a feature vector. This vector is input into a binary Random Forest model to achieve a more comprehensive benign–malignant classification.

All three DL models were trained under the same configuration. A linearly increasing learning rate scheduler was used, with the learning rate gradually increasing from 0 to 0.001. The batch size was set to 8. The AdamW optimizer was employed, and all networks were initialized with ImageNet pre-trained weights. Training lasted for 40 epochs, using BCEWithLogitsLoss() as the loss function, ReLU as the activation function, and L2 regularization to prevent overfitting. During the prediction phase, the model outputs logits, which are mapped to probabilities in the range of 0–1 using the sigmoid function. Input images were center-cropped to 512 × 512 pixels. Data augmentation strategies included: (1) horizontal flipping to increase image diversity; (2) random rotation within ±30° and scaling from 1 to 1.2 to introduce scale variation; (3) brightness adjustment within ±0.25 and image distortion parameters of ±0.15 with a probability of 0.15, simulating potential variations in brightness and distortion during actual imaging. These augmentation settings were designed to enhance model generalization and achieve more robust real-world performance.

**Figure 3:**
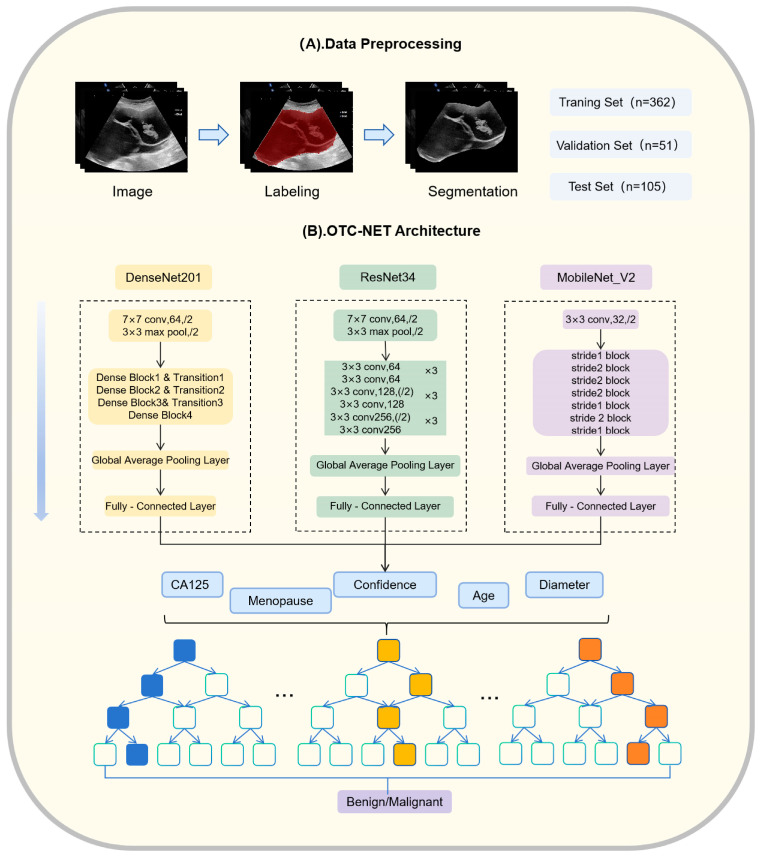
OTC-NET architecture and data preprocessing.

**Figure 4:**
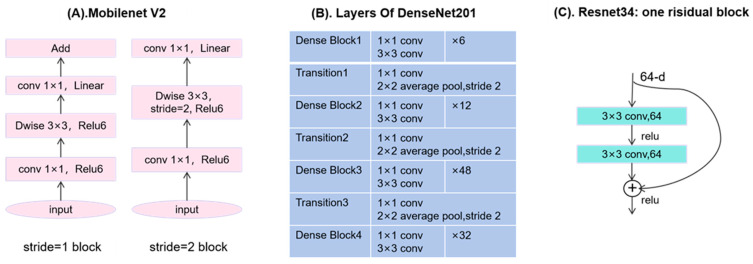
(**A**) Structure of MobileNet_V2 when the stride is 1 or 2. (**B**) Four dense blocks and 3 transition layers in DenseNet201. (**C**) One residual block of ResNet34.

The Random Forest model was trained and tuned using cross-validation. The cross-validation was ranked by patient origin. Using cross-validation combined with grid search, multiple hyperparameter combinations were evaluated to select the best configuration for subsequent classification tasks. The specific parameter settings were as follows: number of decision trees set to 5, 10, 15, and 20; maximum depth set to 5, 10, and 15; minimum samples required to split an internal node set to 2, 4, 6, 8, and 10; minimum samples per leaf set to 2, 4, 6, 8, and 10; maximum feature proportion considered at each split set to 0.2, 0.4, 0.6, 0.8, and 1.0. The hyperparameter combination that achieved the best performance in cross-validation was used for training and prediction to ensure robustness and accuracy on unseen data. The experiments were conducted on a Windows 10 platform equipped with an RTX 3060 GPU (10 GB VRAM), using PyTorch (2.1.0+cu121, San Francisco, CA, USA) as the deep learning framework and Python (version 3.8.0, Amsterdam, The Netherlands) as the programming language. OTC-NET architecture and data preprocessing are shown in Figure 3. The partial structure of some model is specifically shown in Figure 4.

### 2.3. Study Methods

Firstly, to evaluate the performance of OTC-NET, this study compared it with seven DL models and radiologists with different levels of experience. The seven DL models included DenseNet201, ResNet34, MobileNet_V2, ResNet101, VGG13, DenseNet121, and EfficientNet_B5 [23,24,25]. The training parameters for these models were consistent with those used in the first stage of OTC-NET. Additionally, three radiologists with different levels of experience independently reviewed the cases in the test set to assess the performance of AI. The three radiologists included: a junior radiologist who completed standardized residency training and holds a junior professional title (hereafter “junior radiologist”); a mid-level radiologist with specialized training and an intermediate title (hereafter “intermediate radiologist”); and a senior radiologist with over 15 years of experience in gynecologic ultrasound and a senior professional title (hereafter “senior radiologist”). During the readings, the radiologists had no access to any other clinical information about the patients. Secondly, to evaluate the clinical assistance value of OTC-NET, the test set cases were randomly reordered and resubmitted to the radiologists. In this second evaluation, the prediction results generated by the OTC-NET system (including predicted categories and their corresponding probabilities) were provided. The radiologists independently reassessed the cases and recorded their final diagnoses.

### 2.4. Statistical Analysis

The evaluation metrics included the area under the receiver operating characteristic curve (AUC), accuracy, sensitivity, specificity, Youden index (YI), Precision-Recall Area Under the Curve (PR-AUC), and NRI. Accuracy indicates the correct prediction rate for all cases, Sensitivity indicates the correct prediction rate for malignant cases, and Specificity indicates the correct prediction rate for benign cases. Youden’s Index combines sensitivity and specificity to evaluate the discriminative ability of the model. PR-AUC is used to evaluate a model’s classification performance on datasets with imbalanced positive and negative classes. NRI is used to assess how much AI assistance improves a radiologist’s patient risk classification. The results of the deep learning models were computed using relevant Python packages. The confidence intervals for the AUC were estimated using the bootstrap method. Differences in AUC were compared using the DeLong test. Differences in sensitivity and specificity were analyzed using McNemar’s test. The false discovery rate (FDR) correction for multiple comparisons was applied to the resulting *p*-values using the Benjamini–Hochberg procedure. *p*-values less than 0.05 were considered statistically significant. Statistical tests were performed using MedCalc^®^ Statistical Software version 23.3.2 (MedCalc Software Ltd., Ostend, Belgium), and other analyses were conducted using SPSS version 26.0 (IBM Corp., Armonk, NY, USA).

## 3. Results

### 3.1. Basic Information of Patients

The baseline characteristics of patients in the training, validation, and test datasets, as detailed in Table 1. The proportions of benign and malignant cases, as well as the age distribution of patients, were relatively balanced across all datasets. An independent samples t-test was conducted to compare clinical variables between the combined training and validation set (trainval) and the test set. The results showed no statistically significant differences in histological type, age, CA125 levels, maximum tumor diameter or menopausal status between the two groups (*p* > 0.05), indicating consistent clinical characteristics across the datasets and ensuring the reliability of subsequent analyses.

### 3.2. OTC-NET’s Performance

Table 2 presents the performance of the OTC-NET model for ovarian masses under different feature sets. The results showed that the set of “CA125, Diameter, Menopause, and Confidence” achieved the best AUC (0.81). Notably, the basic set of “CA125 and Confidence” already achieved a comparable performance (AUC = 0.80) with the highest accuracy (0.752), suggesting that these two features are the core contributors to model prediction. In contrast, the feature of age or menopausal status did not improve model performance and occasionally reduced it, indicating that these variables had limited predictive value.

**Figure 5:**
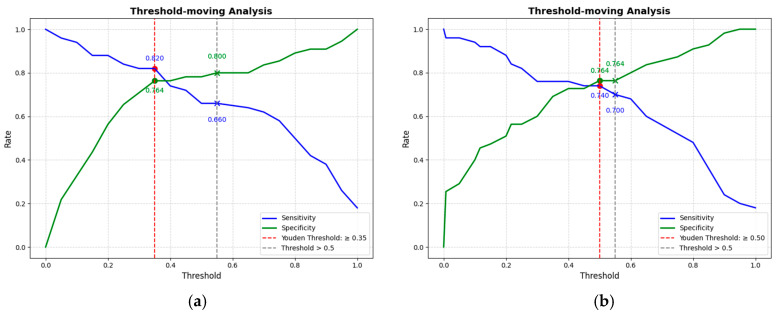
(**a**) The threshold trade-off Curve of the FC1 model. (**b**) The threshold trade-off Curve of the FC2 model. The blue line represents sensitivity, and the green line represents specificity. The red dashed line represents the threshold equal to the optimal Youden’s index, and the gray dashed line represents the threshold equal to the one selected in this paper (>0.5).

**Figure 6:**
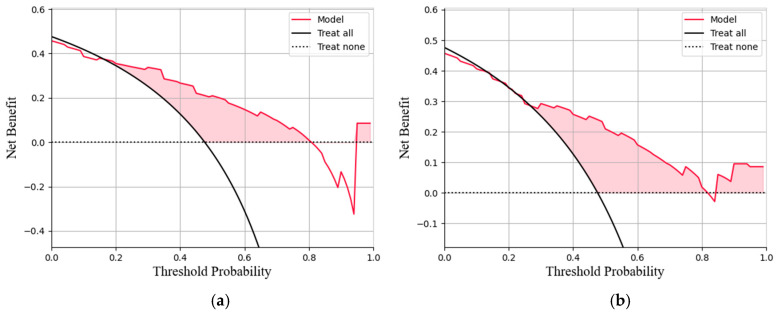
(**a**)The decision curve analysis of the FC1 model. (**b**)The decision curve analysis of the FC2 model. The curve represents the net benefit of using the model to guide clinical decisions at different threshold probabilities, compared with the strategies of treating all or no patients (**b**).

**Figure 7:**
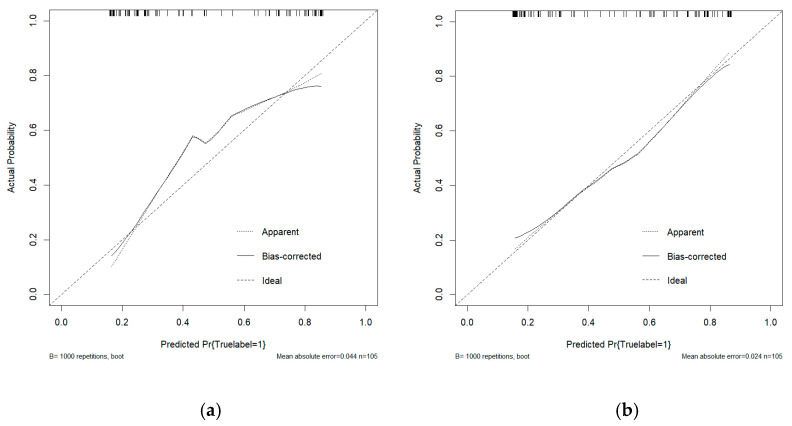
(**a**) The calibration curve analysis of the FC1 model. (**b**) The calibration curve analysis of the FC2 model. The curve demonstrates the agreement between predicted probabilities and observed outcomes, where a curve closer to the diagonal line indicates better calibration.

This study conducted a threshold analysis of the model’s predicted probabilities (Figure 5). The results showed that as the threshold increased, the model’s sensitivity gradually decreased, while its specificity correspondingly increased. The maximum Youden index indicated that the model achieved the optimal balance between sensitivity and specificity at that threshold. For the FC1 model, the optimal threshold was ≥0.35, with a sensitivity of 0.820, specificity of 0.764, and a Youden index of approximately 0.584. For the FC2 model, the threshold was ≥0.05, with a sensitivity of 0.740, specificity of 0.764, and a Youden index of approximately 0.504. An appropriate threshold can be determined to optimize the balance between sensitivity and specificity according to clinical or operational requirements. Figure 6 presents the decision curve analysis (DCA), where the red area represents the potential clinical net benefit that the model could provide across different threshold ranges. When the threshold of the FC1 model is greater than 0.5, the net benefit is 0.210, and when the threshold is greater than or equal to 0.35 (corresponding to the maximum Youden index), the net benefit is 0.324. Similarly, when the threshold of the FC2 model is greater than 0.5, the net benefit is 0.210, and when the threshold is greater than or equal to 0.5 (corresponding to the maximum Youden index), the net benefit is 0.219. Figure 7 shows the calibration curve of the model. The model was further evaluated using the Hosmer–Lemeshow (HL) goodness-of-fit test. The HL test yielded a *p*-value greater than 0.05, suggesting good agreement between the predicted and observed outcomes, indicating that the model was well-calibrated.

### 3.3. DL Model’s Performance

Table 3 presents the experimental results of the deep learning classification models. DenseNet201 achieved the highest AUC at 0.76, compared to 0.73 for ResNet34 and 0.71 for MobileNet_v2. The AUC values of the remaining models were all below 0.70, indicating relatively weaker classification performance. Furthermore, DenseNet201 demonstrated the best balance between sensitivity and specificity, achieving the highest Youden’s index. In contrast, other models showed certain limitations in balancing sensitivity and specificity. For example, DenseNet121 excelled in sensitivity but had relatively low specificity. Therefore, from the perspectives of AUC and Youden’s index, DenseNet201 shows promising potential for clinical application in detecting malignant cases.

### 3.4. Comparison of OTC-NET, DenseNet201, and Radiologists

Table 4 shows the performance of OTC-NET, DenseNet201, and radiologists with varying levels of experience. Three radiologists exhibited distinct diagnostic patterns, characterized by either high sensitivity or high specificity. The intermediate radiologist achieved the highest specificity, significantly higher than the junior (*p* < 0.001) and senior radiologists (*p* < 0.001), but had significantly lower sensitivity than the junior radiologist (*p* < 0.01), suggesting a diagnostic strategy more focused on avoiding false positives. The junior radiologist tended to avoid missed diagnoses, while the senior radiologist achieved the highest accuracy and Youden index by avoiding missed diagnoses.

**Figure 8:**
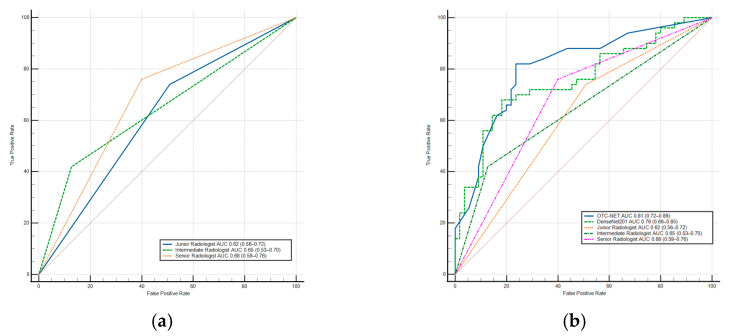
(**a**) The ROC Curve of radiologists. (**b**) The ROC Curve of OTC-NET, DenseNet201, and radiologists. The diagonal lines in both ROC curves represent the results of random guessing and serve as a reference.

DenseNet201 achieved a significantly higher AUC than the junior radiologist (0.76 vs. 0.62, *p* < 0.05) and also outperformed the intermediate and senior radiologists (*p* > 0.05). Additionally, DenseNet201’s sensitivity was significantly higher than the intermediate radiologist (*p* < 0.02). In terms of specificity, DenseNet201 was higher than the junior (*p* < 0.01) and senior radiologists (*p* < 0.03), but slightly lower than the intermediate radiologist (*p* > 0.05). The results show the OTC-NET model demonstrated superior performance, with an AUC significantly higher than the junior (*p* < 0.001), intermediate (*p* < 0.01), and senior radiologists (*p* < 0.05). OTC-NET’s sensitivity was higher than that of the intermediate radiologist (*p* > 0.05), and in terms of specificity, it was significantly superior to the junior radiologist (*p* < 0.01). Figure 8 shows two ROC curve plots: (A) the ROC curve of radiologists; (B) the comparative ROC curves of radiologists, OTC-NET, and DenseNet201.

### 3.5. OTC-NET–Assisted Diagnosis by Radiologists

With AI assistance, the diagnostic performance of junior and senior radiologists improved in terms of AUC and accuracy(Table 5). The junior radiologist’s AUC increased to 0.71 (+0.09, *p* < 0.05), and the senior radiologist’s AUC increased to 0.76 (+0.08, *p* < 0.05), while the intermediate radiologist’s AUC remained unchanged. Accuracy improved by 0.095 and 0.086 for junior and senior radiologists, respectively, with no significant change for the intermediate radiologist. NRI analysis indicated that the junior radiologist experienced the greatest improvement in overall reclassification ability under AI assistance (NRI = 0.180), the senior radiologist showed moderate improvement (NRI = 0.165), and the intermediate radiologist’s NRI was close to zero (0.004), indicating negligible change. Table 6 shows the confusion matrices of radiologists’ diagnoses and improvements with OTC-NET assistance.

### 3.6. Interpretability Analysis

**Figure 9:**
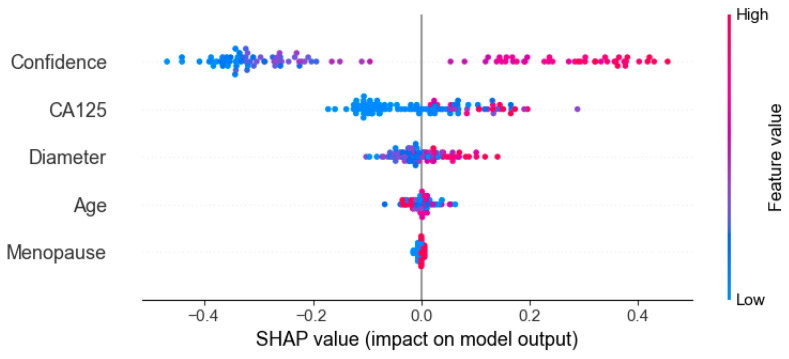
Interpreting the feature contribution to model decisions using SHAP values.

**Figure 10:**
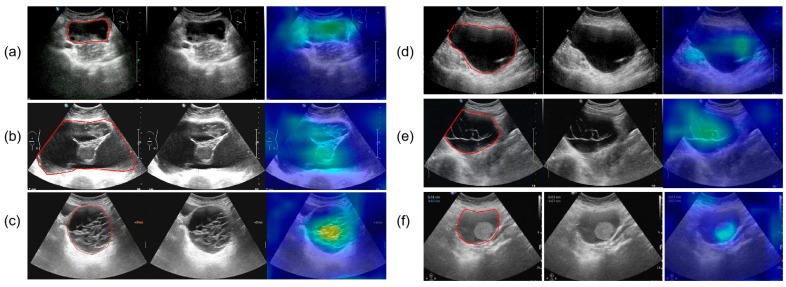
The parts circled in red in the image represent the lesions we labelled. (**a**) A 52-year-old female with high-grade serous carcinoma; (**b**) a 62-year-old female with borderline mucinous cystadenoma with focal intraepithelial carcinoma; (**c**) a 50-year-old female with borderline mucinous tumor; (**d**) a 43-year-old female with mucinous cystadenoma; (**e**) a 26-year-old female with borderline mucinous tumor; (**f**) a 76-year-old female with mucinous cystadenoma.

Figure 9 presents the results of SHAP (SHapley Additive exPlanations), used to analyze the interpretability of the model predictions. The analysis showed that Confidence, CA125 level, and Diameter were the main contributors to distinguishing between benign and malignant cases, while variables such as menopausal status and age contributed relatively little, consistent with the quantitative results shown in Table 2. In the figure, red points on the right indicate that higher feature values increase the probability of predicting malignancy. Figure 10 presents the visualization of the model’s attention distribution on tumors using the Grad-CAM technique. The highlighted regions indicate the areas that contributed most to the model’s discrimination between malignant and benign features. Through comparative analysis, it was found that the model can focus on highly complex regions of the tumor when predicting malignant lesions, whereas for benign lesions, it can attend to the solid regions.

## 4. Discussion

The OTC-NET model achieved its best performance using the feature set “CA125, lesion diameter, menopause, and Confidence,” with an AUC of 0.81. SHAP analysis indicated that Confidence, CA125 level, and Diameter were the primary positive contributors to distinguishing benign from malignant cases, while menopausal status and age contributed minimally to the model’s predictions. By contrast, using only CA125 and Confidence yielded comparable performance (AUC = 0.80). Considering clinical accessibility and experimental performance, this study identifies CA125 and Confidence as the core features for deep learning predictions.

This study also validated the performance of seven deep learning models and three radiologists. Under identical preprocessing methods and training parameters, the seven models exhibited performance differences. Among the seven models, DenseNet201 performed best, achieving an AUC of 0.76. Compared with DenseNet201, OTC-NET performed even better, with an AUC of 0.81. DenseNet201’s AUC was significantly higher than the junior radiologist (*p* < 0.05), the intermediate and senior radiologists (*p* > 0.05). The AUC of OTC-NET was significantly higher than the junior (*p* < 0.001), the intermediate (*p* < 0.01), and the senior radiologist (*p* < 0.05).

This study further examined the effect of OTC-NET assistance on radiologists with different levels of experience. Overall, AI assistance improved the performance of junior and senior radiologists, especially in AUC and accuracy. As the AI assistance in this study was based on the FC1 feature model, which exhibited relatively low sensitivity, radiologists’ sensitivity did not improve significantly with AI assistance, although specificity showed some improvement. The junior radiologist corrected 11 misdiagnoses under AI assistance (all corresponding to correct AI predictions). The senior radiologist corrected 1 malignant and 8 benign cases (all corresponding to correct AI predictions). However, for the intermediate radiologist, AI assistance both corrected 11 originally incorrect diagnoses (10 corresponding to correct AI predictions) and altered 11 previously correct diagnoses (5 corresponding to incorrect AI predictions). This indicates that correct AI predictions can significantly improve diagnostic accuracy, whereas incorrect AI predictions carry a potential risk of misleading radiologists. In this study, junior and senior radiologists were hardly affected by incorrect AI predictions. Continuously ensuring AI tool reliability and educating radiologists about potential AI errors is essential to avoid blind reliance in clinical practice.

OTC-NET identifies multiple features of a case and generates probabilistic classifications through complex algorithms, whereas radiologists rely on professional knowledge and clinical experience to assess lesion malignancy risk [26]. As deep learning models currently lack interpretability and cannot communicate with patients [27], we believe such models cannot replace radiologists in clinical work. Nevertheless, with their rapid computational power and strong feature learning capabilities, they are expected to become clinical decision-support tools, improving diagnostic accuracy [28]. In this study, OTC-NET achieved an AUC of 0.81 using multiple core features, indicating that the model performs relatively reliably in distinguishing benign from malignant cases. It can provide diagnostic guidance for junior and intermediate radiologists, prompting them to conduct secondary reviews or group discussions for cases with inconsistent opinions, thereby reducing the risk of missed or incorrect diagnoses. Furthermore, the model can adjust the threshold to identify patients with a high probability of malignancy, thereby helping radiologists prioritize and focus on these cases, enhancing the efficiency and accuracy of medical services. Compared with single deep learning models or diagnoses from individual radiologists, OTC-NET integrates multi-source information to generate probabilistic outputs, improving both AUC and overall diagnostic accuracy. This further underscores the value of leveraging large-scale datasets and multimodal information in model development.

This study still has several limitations. Firstly, the dataset was limited in size and lacked prospective validation, which may affect the model’s generalizability. Secondly, during the dataset collection phase, sonographers did use Doppler information when performing O-RADS classification; however, Doppler images were not utilized during model construction, training, or the physician-involved testing phase, which may have limited the information available for the model to learn. In the future, we plan to further investigate incorporating blood flow signal grading as an additional feature into the model development.

## 5. Conclusions

This study proposes a multimodal classification method, OTC-NET, which can integrate multiple types of features to further improve the accuracy of O-RADS 4 ovarian tumor classification. Compared with seven deep learning models and three radiologists of varying experience levels, OTC-NET demonstrated superior performance, achieving an AUC of 0.81 (95% CI: 0.72–0.89), an accuracy of 0.733, a sensitivity of 0.660, and a specificity of 0.800. Its performance in terms of AUC and accuracy surpassed both deep learning models and senior radiologists. Further experiments showed that OTC-NET-assisted diagnosis effectively improved the overall diagnostic performance of junior and senior radiologists (*p* < 0.05). In summary, OTC-NET exhibits excellent diagnostic performance in the benign–malignant classification of O-RADS 4 ovarian tumors, demonstrating strong clinical applicability.

## Figures and Tables

**Table 1:** Baseline characteristics of patients in the training, validation, and test sets.

Characteristic	Training	Validation	Test	*p* Value
No. of patients	362	51	105	
Histological type				0.930
Benign	187 (51.7)	26 (51.0)	55 (52.4)	
Malignant	175 (48.3)	25 (49.0)	50 (47.6)	
Age at diagnosis (y)				0.341
Mean ± SD	47 ± 15	47 ± 13	47 ± 17	
Range	13–89	12–73	11–81	
Largest lesion diameter (mm)				0.217
Mean ± SD	124.9 ± 70.7	119.6 ± 59.7	129.7 ± 74.6	
Range	16–600	25–303	25–526	
CA125 (U/mL)				0.236
>35	169 (46.7)	23 (45.1)	46 (43.8)	
≤35	193 (53.3)	28 (54.9)	59 (56.2)	
Menopausal status				0.422
Premenopausal	205 (56.6)	25 (49.0)	55 (52.4)	
Postmenopausal	157 (43.4)	26 (51.0)	50 (47.6)	

**Table 2:** Different feature combinations yield different results.

Feature Set		AUC (95% CI)	Accuracy	Sensitivity	Specificity
FC1	CA125, Diameter, Menopause, Confidence	0.81 (0.72–0.89)	0.733	0.660	0.800
FC2	CA125, Confidence	0.80 (0.72–0.89)	0.733	0.700	0.764
FC3	CA125, Menopause, Confidence	0.80 (0.72–0.89)	0.724	0.640	0.800
FC4	CA125, Diameter, Confidence	0.79 (0.71–0.88)	0.714	0.640	0.782
FC5	CA125, Age, Diameter, Menopause, Confidence	0.78 (0.69–0.87)	0.695	0.580	0.800
FC6	Age, Confidence	0.76 (0.66–0.85)	0.714	0.560	0.855
FC7	Menopause, Confidence	0.75 (0.66–0.84)	0.667	0.560	0.764
FC8	Diameter, Confidence	0.74 (0.65–0.83)	0.724	0.660	0.782

**Table 3:** The diagnostic performance of the Neural Network Classification Model.

Module	AUC (95%CI)	Accuracy	Sensitivity	Specificity	YI
DenseNet201	0.76 (0.66–0.85)	0.733	0.640	0.818	0.458
ResNet34	0.73 (0.63–0.83)	0.648	0.520	0.764	0.284
MobileNet_V2	0.71 (0.61–0.80)	0.686	0.640	0.727	0.367
ResNet101	0.70 (0.59–0.80)	0.629	0.500	0.745	0.245
VGG13	0.70 (0.58–0.80)	0.657	0.640	0.672	0.312
DenseNe121	0.69 (0.58–0.79)	0.619	0.740	0.509	0.249
EfficientNet_B5	0.68 (0.57–0.77)	0.610	0.680	0.545	0.225

**Table 4:** Diagnostic performance of OTC-NET, DenseNet201, and radiologists with different experience levels.

	AUC (95% CI)	PR-AUC	Accuracy	Sensitivity	Specificity	YI
OTC-NET	0.81 (0.72–0.89) ^abc^	0.795	0.733	0.660	0.800 ^a^	0.460
DenseNet201	0.76 (0.66–0.85) ^a^	0.767	0.733	0.640 ^b^	0.818 ^ac^	0.458
Senior Radiologist	0.68 (0.59–0.76)	0.611	0.676	0.760 ^b^	0.600 ^b^	0.360
Intermediate Radiologist	0.65 (0.53–0.70)	0.638	0.657	0.420 ^ac^	0.873 ^ac^	0.293
Junior Radiologist	0.62 (0.56–0.72)	0.555	0.610	0.740 ^b^	0.491 ^b^	0.231

^a^ *p* < 0.05, compared with Junior Radiologist; ^b^ *p* < 0.05, compared with Intermediate Radiologist; ^c^ *p* < 0.05, compared with Senior Radiologist.

**Table 5:** The diagnosing performance of radiologists and the improvement with OTC-NET Assistance. Arrows (↑) have been added next to the values that show an increase.

AI-Assisted						
Radiologist	AUC (95% CI)	PR-AUC	Accuracy	Sensitivity	Specificity	NRI
Junior Radiologist	0.71 (0.61–0.79)	0.645	0.705	0.720	0.691	0.180
	(+0.090) ↑	(+0.090) ↑	(+0.095) ↑	(−0.020)	(+0.200) ↑	
Intermediate Radiologist	0.65 (0.57–0.73)	0.629	0.657	0.460	0.836	0.004
	(0.00)	(−0.009)	(0.00)	(+0.040) ↑	(−0.037)	
Senior Radiologist	0.76 (0.69–0.85)	0.701	0.762	0.780	0.745	0.165
	(+0.080) ↑	(+0.090) ↑	(+0.086) ↑	(+0.020) ↑	(+0.145) ↑	

**Table 6:** Confusion matrices of radiologists’ diagnoses and improvements with OTC-NET assistance.

		Prediction	Benign (Before AI)	Malignant (Before AI)	Benign (AI-Assisted)	Malignant (AI-Assisted)
	True					
Junior Radiologist	Benign		27	28	38	17
	Malignant		13	37	14	36
Intermediate Radiologist	Benign		48	7	46	9
	Malignant		29	21	27	23
Senior Radiologist	Benign		33	22	41	14
	Malignant		12	38	11	39

## Data Availability

The datasets used and analyzed during the current study are available from the corresponding author on reasonable request. The code is publicly available at https://github.com/chen1147599383-sys/OTCNET22.git (accessed on 25 October 2025).

## Abbreviations

The following abbreviations are used in this manuscript:

O-RADS	Ovarian-Adnexal Reporting and Data System
US	Ultrasonography
AUC	Area under the receiver operating characteristic curve
DL	Deep Learning
YI	Youden’s index.
AI	Artificial intelligence

## References

[1] Han B., Zheng R., Zeng H., Wang S., Sun K., Chen R., Li L., Wei W., He J. (2024). Cancer incidence and mortality in China, 2022. J. Natl. Cancer Cent..

[2] Baker V.V. (2001). Treatment Options for Ovarian Cancer. Clin. Obstet. Gynecol..

[3] Terzic M., Rapisarda A.M.C., Della Corte L., Manchanda R., Aimagambetova G., Norton M., Garzon S., Riemma G., King C.R., Chiofalo B. (2021). Diagnostic work-up in paediatric and adolescent patients with adnexal masses: An evidence-based approach. J. Obstet. Gynaecol..

[4] Sadowski E.A., Stein E.B., Thomassin-Naggara I., Rockall A., Nougaret S., Reinhold C., Maturen K.E. (2023). O-RADS MRI After Initial Ultrasound for Adnexal Lesions: AJR Expert Panel Narrative Review. Am. J. Roentgenol..

[5] Andreotti R.F., Timmerman D., Strachowski L.M., Froyman W., Benacerraf B.R., Bennett G.L., Bourne T., Brown D.L., Coleman B.G., Frates M.C. (2020). O-RADS US risk stratification and management system: A consensus guideline from the ACR Ovarian-Adnexal Reporting and Data System Committee. Radiology.

[6] Vara J., Manzour N., Chacon E., Lopez-Picazo A., Linares M., Pascual M.A., Guerriero S., Alcazar J.L. (2022). Ovarian Adnexal Reporting Data System (O-RADS) for classifying adnexal masses: A systematic review and meta-analysis. Cancers.

[7] Lee S., Lee J.E., Hwang J.A., Shin H. (2023). O-RADS US: A Systematic Review and Meta-Analysis of Category-specific Malignancy Rates. Radiology.

[8] Fleming N.D., Westin S.N., Meyer L.A., Shafer A., Rauh-Hain J.A., Onstad M., Cobb L., Bevers M., Fellman B.M., Burzawa J. (2021). Correlation of surgeon radiology assessment with laparoscopic disease site scoring in patients with advanced ovarian cancer. Int. J. Gynecol. Cancer.

[9] Buranaworathitikul P., Wisanumahimachai V., Phoblap N., Porngasemsart Y., Rugfoong W., Yotchana N., Uthaichalanont P., Jiampochaman T., Kunanukulwatana C., Thiamkaew A. (2024). Accuracy of O-RADS System in Differentiating Between Benign and Malignant Adnexal Masses Assessed via External Validation by Inexperienced Gynecologists. Cancers.

[10] Timmerman D., Ameye L., Fischerova D., Epstein E., Melis G.B., Guerriero S., Van Holsbeke C., Savelli L., Fruscio R., Lissoni A.A. (2010). Simple ultrasound rules to distinguish between benign and malignant adnexal masses before surgery: Prospective validation by IOTA group. Bmj.

[11] Wang J., Zhu H., Wang S.-H., Zhang Y.-D. (2021). A review of deep learning on medical image analysis. Mob. Netw. Appl..

[12] Suganyadevi S., Seethalakshmi V., Balasamy K. (2022). A review on deep learning in medical image analysis. Int. J. Multimed. Inf. Retr..

[13] Chen H., Yang B.-W., Qian L., Meng Y.-S., Bai X.-H., Hong X.-W., He X., Jiang M.-J., Yuan F., Du Q.-W. (2022). Deep learning prediction of ovarian malignancy at US compared with O-RADS and expert assessment. Radiology.

[14] Christiansen F., Epstein E., Smedberg E., Åkerlund M., Smith K., Epstein E. (2021). Ultrasound image analysis using deep neural networks for discriminating between benign and malignant ovarian tumors: Comparison with expert subjective assessment. Ultrasound Obstet. Gynecol..

[15] Gao Y., Zeng S., Xu X., Li H., Yao S., Song K., Li X., Chen L., Tang J., Xing H. (2022). Deep learning-enabled pelvic ultrasound images for accurate diagnosis of ovarian cancer in China: A retrospective, multicentre, diagnostic study. Lancet Digit. Health.

[16] Xiang H., Xiao Y., Li F., Li C., Liu L., Deng T., Yan C., Zhou F., Wang X., Ou J. (2024). Development and validation of an interpretable model integrating multimodal information for improving ovarian cancer diagnosis. Nat. Commun..

[17] Ruan Y., Liu Z., Yang Y., Feng L., Liu P. A Comparative Study of Deep Learning Models and Expert Diagnosis in benign and malignant Classification of Ovarian Masses Based on Ultrasound Images. Proceedings of the 2025 International Conference on Health Big Data.

[18] Severinski K., Cvija T. (2021). Medical data annotation and json to dataset conversion using LabelMe and Python. Ri-STEM-2021.

[19] Huang G., Liu Z., Van Der Maaten L., Weinberger K.Q. Densely connected convolutional networks. Proceedings of the IEEE Conference on Computer Vision and Pattern Recognition.

[20] Xu W., Fu Y.-L., Zhu D. (2023). ResNet and its application to medical image processing: Research progress and challenges. Comput. Methods Programs Biomed..

[21] Sandler M., Howard A., Zhu M., Zhmoginov A., Chen L.-C. Mobilenetv2: Inverted residuals and linear bottlenecks. Proceedings of the IEEE Conference on Computer Vision and Pattern Recognition.

[22] Salman H.A., Kalakech A., Steiti A. (2024). Random forest algorithm overview. Babylon. J. Mach. Learn..

[23] Zhang Q. (2022). A novel ResNet101 model based on dense dilated convolution for image classification. SN Appl. Sci..

[24] Tan M., Le Q. Efficientnet: Rethinking model scaling for convolutional neural networks. Proceedings of the 36th International Conference on Machine Learning.

[25] Simonyan K., Zisserman A. (2014). Very deep convolutional networks for large-scale image recognition. arXiv.

[26] Strachowski L.M., Jha P., Phillips C.H., Blanchette Porter M.M., Froyman W., Glanc P., Guo Y., Patel M.D., Reinhold C., Suh-Burgmann E.J. (2023). O-RADS US v2022: An update from the American College of radiology’s ovarian-adnexal reporting and data system US committee. Radiology.

[27] ŞAHiN E., Arslan N.N., Özdemir D. (2025). Applications. Unlocking the black box: An in-depth review on interpretability, explainability, and reliability in deep learning. Neural Comput. Appl..

[28] Gou F., Liu J., Xiao C., Wu J. (2024). Research on artificial-intelligence-assisted medicine: A survey on medical artificial intelligence. Diagnostics.

